# A novel approach to identifying and quantifying neutrophil extracellular trap formation in septic dogs using immunofluorescence microscopy

**DOI:** 10.1186/s12917-018-1523-z

**Published:** 2018-06-27

**Authors:** Ronald H. L. Li, Lynelle R. Johnson, Casey Kohen, Fern Tablin

**Affiliations:** 1 Department of Veterinary Surgical and Radiological Sciences, School of Veterinary Medicine, University of California, One Shield Avenue, Davis, California 95161 USA; 2 Department of Medicine and Epidemiology, School of Veterinary Medicine, University of California, Davis, California USA; 3 William Pritchard Veterinary Medical Teach Hospital, University of California, Davis, California USA; 4 Department of Anatomy, Physiology and Cell Biology, School of Veterinary Medicine, University of California, Davis, California USA

**Keywords:** Peptidylarginine deiminase, Citrullinated histones, Cell-free DNA, Sepsis

## Abstract

**Background:**

Canine neutrophils release neutrophil extracellular traps (NETs) in response to lipopolysaccharide but NETs from clinical septic dogs had not been identified. The primary aim is to describe the methodology of identifying and quantifying neutrophil extracellular traps (NETs) in cytology samples of septic foci in dogs with sepsis using immunofluorescence microscopy. Cytology samples including endotracheal tracheal wash (ETW), bronchoalveolar lavage (BAL), abdominal and pleural effusion collected from 5 dogs (3 septic, 2 non-septic) were fixed, permeabilized and stained for myeloperoxidase (MPO), citrullinated histone H3 (citH3) and cell-free DNA (cfDNA). Fluorescence microscopy was used to identify and quantify NETs in 10 random views at 40× magnification. NETs were identified based on co-localization of MPO, citH3 and cfDNA. NETs were quantified as a ratio (number of NETs: number of neutrophils). Neutrophils were identified based on cytoplasmic MPO, cellular diameter and nuclear morphology.

**Results:**

NETs were identified and quantified in all cytology samples collected from septic dogs. A small number of NETs was documented in one dog with sterile chronic bronchitis. No NETs were found in sterile abdominal effusion collected from one dog with congestive heart failure.

**Conclusions:**

Immunofluorescence microscopy could be a useful tool for the study of NETs in dogs with clinical sepsis.

## Background

In response to bacterial infection, neutrophils release neutrophil extracellular traps (NETs), which are web-like scaffolds of extracellular DNA decorated with histones and neutrophil granular proteins such as myeloperoxidase (MPO) and neutrophil elastase [1]. Bacteria, entrapped within NETs, are either phagocytosed by adjacent neutrophils or killed by high concentrations of extracellular antimicrobial proteins [2]. Although NETs are found to limit bacterial dissemination in septic mice, overzealous NET production (NETosis) is associated with sepsis-associated organ dysfunction such as acute lung injury, acute kidney injury and mortality [3, 4]. This paradox highlights the fact that dysregulation of NETosis likely plays a key role in perpetuating tissue damage and organ dysfunction. NET components such as histones, once released extracellularly, can function as a damage-associated molecular pattern resulting in cytotoxicity, inflammation and microvascular dysfunction [5]. In human septic patients, elevated intravascular NET components such as cell-free DNA (cfDNA) and citrullinated histones are associated with mortality [6, 7].

To date, NETs have not been documented in dogs with bacterial sepsis. Isolated canine neutrophils have been shown to release NETs in response to *E. coli* lipopolysaccharide and the chemical, phorbol 12-myristate 13-acetate [8, 9]. Several methods of plasma cfDNA quantification have been described in dogs with sepsis, neoplasia and immune-mediated haemolytic anemia [8, 10]. However, the use of cfDNA as a NETosis biomarker is controversial as there is evidence suggesting that cfDNA does not originate specifically from NET-producing neutrophils [11]. Citrullinated histones and myeloperoxidase (MPO), other NET biomarkers, have not been identified in septic dogs. Because NETs may contribute to the pathophysiology of sepsis in dogs, a method allowing specific and objective identification and quantification of NETs in septic dogs is needed.

In this methodology manuscript, we sought to describe a novel technique to visualize and quantify NETs in cytological samples of septic foci in dogs using immunofluorescence microscopy.

## Methods

The study protocol was approved by the Institutional Animal Care and Use Committee at the University of California, Davis. Client-owned dogs admitted to the William R. Pritchard Veterinary Medical Teaching Hospital of University of California, Davis and diagnosed with sepsis were eligible for enrolment. Sepsis was diagnosed based on the presence of systemic or local bacterial infection and systemic inflammatory response syndrome (SIRS). SIRS was identified based on previously published criteria [12]. Only dogs with cytological samples collected from their respective septic foci were enrolled. Clinical management of patients was determined by the primary attending clinicians. Bacterial infection was diagnosed based on cytologic identification of intracellular bacteria and positive bacterial cultures from patient samples. Non-septic dogs with matched cytology samples were included as controls. Medical records were reviewed for patients’ signalment including age, breed and sex as well as outcome and bacterial culture results.

In Dog 1, an ETW was performed by passing an infant feeding tube down a sterile endotracheal tube to approximately the level of the carina. In Dog 4, bronchoalveolar lavage (BAL) at multiple sites (right cranial, left and right caudal bronchi) was performed through the biopsy channel of the endoscope after bronchoscopic examination. Abdominal and pleural effusion were collected via ultrasound-guided needle aspiration.

Cytology samples were immediately placed on ice and diluted (1-fold) in Dulbecco’s phosphate buffered saline (DPBS) with 1% bovine serum albumin. Samples were further diluted (2 to 3-fold) before concentrated onto microscope slides using a cytocentrifuge (Cytospin 4, ThermoScientific Inc., Grand Island, NY) (1500 rpm, 5 min), air dried, and stored at -20 °C until further analysis. The residual samples were submitted to the University of California Veterinary Medical Teaching Hospital clinical diagnostic laboratory for culture and susceptibility, total nucleated cell counts (Advia 120, Siemens, Deerfield, IL) and cytologic assessment by board-certified clinical pathologists.

## Immunofluorescence microscopy

**Fig. 1:**
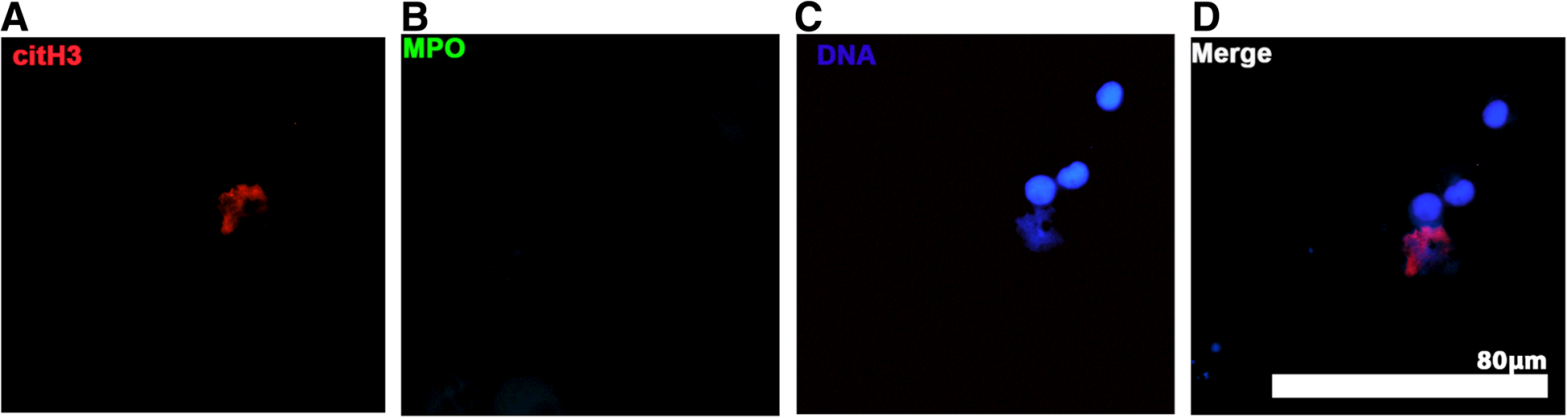
Representative immunofluorescent images of cytology samples of bronchoalveolar lavage from a dog demonstrating the use of interference controls. Cells were fixed, permeabilized, and incubated with rabbit anti-human citrullinated histone H3 (citH3) antibody, followed by secondary goat anti-rabbit antibody conjugated with Alexa Fluor 568. After blocking with rabbit serum and unconjugated goat anti-rabbit Fab fragments, cells were incubated with goat anti-rabbit antibody conjugated with Alexa Fluor 488 without the second primary antibody against myeloperoxidase (MPO). Cells analysed under the TEXAS RED channel demonstrated presence of citH3 (red) (**a**), while no non-specific binding of secondary body conjugated to Alexa Fluor 488 was detected (**b**). DNA  was stained with DAPI (**c**) to ensure the colocalization of citH3 and DNA (**d**). Original 40× magnification

Cells were first fixed in 4% paraformaldehyde then permeabilized using 1% NP-40 (Surfact-AMPs™ NP-40, Pierce, Rockford, IL) before washing 3 times with DPBS. A modified double immunolabeling protocol was utilized because the primary antibodies used to detect MPO and citrullinated histone H3 (citH3) were produced in the same species [13]. In brief, cells were first blocked with 5% goat serum (1 h, 37 °C) before incubating with 1:400 (2.5 μg/ml) rabbit polyclonal anti-citH3 antibodies (ab5103, Abcam, Cambridge, MA) (1 h, 37 °C). After washing (DPBS, 3 times), secondary antibodies (1:200, 10 μg/ml, polyclonal goat anti-rabbit antibodies conjugated with Alexa Fluor 568, A-11011, ThermoFisher Scientific, Rockford, IL) was added to cells and incubated for 1 h at 37 °C. Blocking steps utilizing 10% rabbit serum (4 °C, overnight) followed by incubation with unconjugated goat anti-rabbit Fab fragments (50 μg/mL, Jackson ImmunoResearch Laboratories, West Grove, PA) for 2 h at room temperature were performed to prevent the MPO primary antibody from binding to the first secondary antibody. To identify MPO, samples were labelled with polyclonal rabbit anti-human MPO antibody (1:200, 2 μg/ml, A 0398, Dako, Denmark) for 1 h at 37 °C followed by a secondary antibody (1:200, 10 μg/ml, Alexa Fluor 488 conjugated polyclonal goat anti-rabbit IgG, A-11008, ThermoFisher Scientific, Rockford, IL). DNA was stained with 300 nM 4′,6-Diamidino-2-Phenylindole, Dihydrochloride. Interference controls without either primary antibodies in the second immunolabeling step (Type II inference) were demonstrated in Fig. 1 [13].

**Fig. 2:**
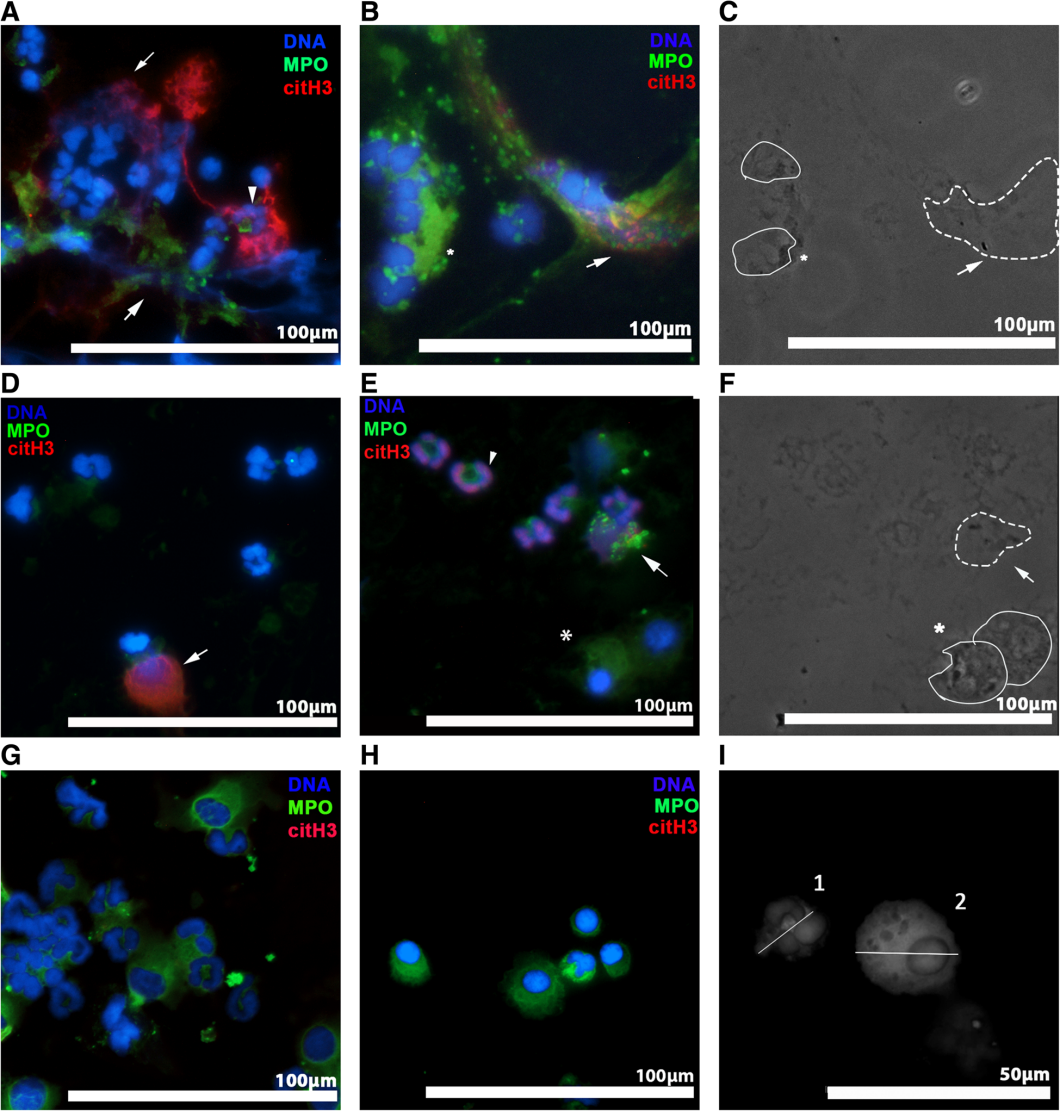
Representative immunofluorescent images of cytology samples from 3 septic and 2 non-septic dogs. Cells were fixed, permeabilized and stained for citrullinated histone H3 (citH3, red), myeloperoxidase (MPO, green) and DNA (blue). NETs were identified by co-localization of decondensed cell-free DNA (cfDNA), citH3 and MPO. **a**, **b** Endotracheal wash from a dog with aspiration pneumonia. Note the vast amount of decondensed DNA decorated with extracellular citH3 and MPO surrounding nearby neutrophils (dotted outline). Activated pulmonary macrophages also were identified (*). **c** In the respective phase contrast image, phagocytized bacteria within activated pulmonary macrophages (*) and bacteria (arrow) within a NET (dash outline) were detected. NETs (dotted outline) were identified in septic abdominal (**d**) and pleural fluid (**e**). **e**, **f** The respective phase contrast image showed bacteria within a NET (dotted outline) and phagocytized bacteria within macrophages (*). Some neutrophils in septic effusion (**a**, **e**) had chromatin stained positive for citH3 (arrow heads). Non-degenerate neutrophils and activated macrophages were seen in abdominal fluid acquired from a dog with congestive heart failure (**g**) and a dog with chronic bronchitis (**h**); no NETs were identified. **i** A grayscale image stained for MPO and DNA demonstrating the measurement of cell diameter and nuclear morphology. Diameter of cell 1 was measured to be 8 μm. Cell 1 was identified to be a neutrophil based on its lobulated nuclei. Cell 2 was identified to be an activated pulmonary macrophage with a cell diameter of 18.5 μm, with rounded non-lobulated nuclei and presence of cytoplasmic vacuoles. Original 40× magnification. **a**–**h** Scale bar = 100 μm; (**i**) Scale bar = 50 μm

An EVOS FL Cell Imaging System (ThermoFisher Scientific, Rockford, IL) was used to acquire the immunofluorescent images. Prior to image capture and analysis, the authors were blinded to the conditions by randomly assigning a number to each slide. At 40× magnification, 10 random fields were captured for each sample and the images were acquired and analyzed in a blinded manner using available software (ImageJ, v1.50 g, National Institute of Health). Exposure times of each channel (blue, green or red) were kept constant throughout the analysis. Neutrophils were identified based on nuclear morphology, cell diameter of less than 15 μm (Fig. 2i) and presence of MPO within the cytoplasm. NETs were identified based on co-localization of cfDNA, extracellular MPO, and citH3 [14]. The quantity of NETs in each slide was expressed as a ratio (number NETs: number of neutrophils) in 10 random fields. Cells with citH3 expression were quantified as a ratio (Cells _CitH3_: Cells _no CitH3_) by dividing the total of number of cells expressing intracellular citH3 by the total number of cells without intracellular citH3. For septic samples, phase-contrast microscopy was used to visualize bacteria.

## Results

Five dogs (3 with sepsis, 2 without sepsis) were enrolled between February and December 2016. Initial presenting signs and other clinical parameters are summarized in Table 1. All dogs survived to discharge, except Dog 3, which underwent cardiopulmonary arrest shortly after presentation.

**Table 1:** Summary of demographic variables, presenting signs and clinical parameters

				SIRS criteria				
Dog	Age	Breed	Presenting Signs	HR (per min)	RR (per minute)	Body temperature (°C)	WBC (× 10^3^/μL)	Diagnosis
1	11	Pug	Acute onset of respiratory distress following 24 h of treatment for presumed aspiration pneumonia	60	60	37.2	27.3	Respiratory failure secondary to aspiration pneumonia
2	2	Mixed	Acute onset of respiratory distress with right-sided pneumothorax and small-volume pleural effusion	84	60	39.4	22.0	Bacterial pyothorax
3	9	German Shepherd Dog	Chronic lethargy, inappetence and abdominal pain	150	36	43.5	9.72	Septic peritonitis due to gastric wall necrosis
4	8	Brittany Spaniel	Intermittent productive cough	114	30	38.9	18.3	Chronic bronchitis
5	14	Newfoundland	1-week history of cough and abdominal swelling Atrial fibrillation	240	40	38.1	N/A	Congestive heart failure

*HR* heart rate

*RR* respiratory rate

*WBC* white blood count

*N/A* not available

Cytology and bacterial culture results, as well as NET and citH3 quantification are summarized in Table 2. Immunofluorescent images revealed the presence of NETs in the samples from septic dogs including ETW (Fig. 2a, b), abdominal fluid (Fig. 2d) and pleural effusion (Fig. 2e). NETs consisted of extracellular DNA, MPO and citH3. Using phase contrast microscopy of the respective immunofluorescent views, intracellular bacteria were noted within neutrophils and/or macrophages as well as within NETs (Fig. 2c, f). The quantity of NETs was 3-fold higher in septic ETW compared to that in BAL of chronic bronchitis (Table 2). Although an abundance of non-degenerate neutrophils was found in sterile abdominal effusion obtained from the dog with congestive heart failure, no NETs were identified (Fig. 2g). A subset of neutrophils from septic effusions also underwent intracellular histone H3 citrullination (Fig. 2a, e), whereas intracellular citH3 was rarely observed in cytology obtained from dogs without bacterial infections (Fig. 2g, h) (Table 2).

**Table 2:** Summary of cytology, bacteriology results and NET quantification

	Differential cytology	Cytological diagnosis	Bacteriology	NET (NET:cells)	Ratio of intracellular citH3
Endotracheal Tracheal Lavage					
Dog 1	TNCC: 10.36 × 10^3^/μL Neutrophils: 85% Lymphocytes: 0% Macrophages: 15% Eosinophils: 0%	Marked suppurative inflammation	*Staphylococcus intermedius, Corynebacterium sp., Mycoplasma sp.*	0.070	0.093
Pleural effusion					
Dog 2	TNCC: 482 × 10^3^ /μL Neutrophils: 78% Macrophages: 20% Others: 2%	Marked septic suppurative inflammation	*Streptococcus Viridans* *Fusobacterium nucleatum*	0.26	0.67
Abdominal Fluid					
Dog 3	TNCC: 21.6 × 10^3^/μL Neutrophils: 84% Lymphocytes:12% Macrophages: 4% Eosinophils: 0%	Moderate septic suppurative inflammation	*Enterococcus faecalis* *Enterococcus faecium*	0.14	0.24
Bronchoalveolar Lavage					
Dog 4	TNCC: 0.54 × 10^3^/μL Neutrophils: 17% Lymphocytes: 18% Macrophages: 41% Eosinophils: 24%	Mild mixed inflammation	*Negative*	0.016	0.032
Pleural Effusion					
Dog 5	TNCC: 2.5 × 10^3^/μL Neutrophils: 28% Lymphocytes: 1% Macrophages: 69% Others: 2%	Mild mixed inflammation	*Negative*	0	0.0032

*TNCC* total nucleated cell count

*NET* neutrophil extracellular trap

*CitH3* citrullinated histone H3

## Discussion

By utilizing double immunolabelling and 3-channel fluorescence microscopy, we identified, for the time first, NET formation in dogs with clinical sepsis. We also developed a protocol for quantifying NETs and intracellular citH3 expression in clinical cytology samples using accessible public domain software.

In this study, NETs from cytology samples were quantified based on co-localization of NET components: cfDNA, MPO and citH3. MPO, which resides within neutrophil primary granules, catalyzes the formation of hypochlorite and is released into phagosomes where bacteria are killed by high concentrations of hypochlorite and peroxides [15]. During NETosis, granular proteins including MPO along with cfDNA, are released into the extracellular space [16]. However, since leakage of MPO and DNA also occurs in necrosis, cfDNA and extracellular MPO are not specific markers of NETosis. Histone citrullination or deimination, which converts the N-terminus arginine to citrulline, is a unique process occurring in neutrophils undergoing NETosis [17]. Histone citrullination, catalzyed by the enzyme, peptidylarginine deiminase 4 (PAD4), alters the electrostatic interactions between DNA and histones causing chromatin decondensation and release of cfDNA during NETosis [18]. We have recently shown that lipopolysaccharide-mediated NETosis in vitro requires histone H3 hypercitrullination by PAD in canine neutrophils [9]. The substantial number of citH3 positive cells identified in culture-positive samples suggests that a similar cellular process also occurs in vivo. Thus, citH3 within canine NETs may serve as diagnostic marker of bacterial infection in dogs.

The discovery of NETs in septic foci highlights that NETosis is likely a highly-conserved component of innate immunity during bacterial infections in dogs as in other species. We provided, for the first time, direct imaging evidence for the binding of bacteria within the structural elements of canine NETs. This, however, does not definitively indicate that NETs in dogs can entrap bacteria and prevent systemic dissemination of bacteria in vivo. Based on other in vitro and in vivo experiments, bacteria like *Staphylococcus aureus* can acquire resistance to the bactericidal properties of NETs over time [19]. An added level of complexity in the study of NETs is that while NETs possess important antimicrobial properties, overzealous production of NETs can be detrimental to the host [4]. MPO can augment tissue inflammation by activating nearby neutrophils and inducing further NETosis [16, 20]. Free histones have been shown to act as damage-associated molecular patterns impairing endothelial function and initiating inflammation, cytotoxicity and coagulation in mice [5, 21]. It is unknown if citH3 released via NETosis within body compartments could lead to elevated circulating levels of citH3 in dogs. CitH3 as a biomarker of canine sepsis requires further investigation.

The abundance of NETs found in septic tracheal wash fluid from Dog 1 suggests that canine neutrophils release NETs into the airways in response to acute bacterial infection. However, a small quantity of NETs also was found in BAL from Dog 4 with sterile chronic pulmonary inflammation. In human beings, NETosis is commonly associated with chronic bronchitis and chronic obstructive pulmonary disease. The degree of NETosis correlates with the presence of neutrophils in the airways, and it is conceivable that NETosis also occurs in cases of canine chronic bronchitis with progressively severe neutrophilic inflammation [22]. This requires further investigation in a larger number of dogs.

This study has several limitations. Direct visualization and quantification of NETs in cytology samples can be challenging due to the lack of standardized cell counts and variable cell types. Because the number of NETs is dependent on the number of neutrophils in each sample, we measured NETs and citH3 positive neutrophils relative to the number of neutrophils. Due to the presence of various cell types, we also employed robust criteria to classify and quantify neutrophils. However, canine neutrophils that have not yet released NETs but have undergone chromatin decondensation may have non-lobulated and rounded nuclei [17]. These changes in chromatin morphology might have affected our ability to accurately identify neutrophils and hence under- or overestimated the quantity of NETs produced. Co-immunostaining of a canine-specific neutrophil surface marker with a different fluorophore could allow for more accurate cell enumeration. Because microscopic evaluation is subjected to observer bias, we utilized a standardized protocol to acquire and analyze images in a randomized and blinded manner. A major drawback of this technique is that it is labor intensive, requiring multiple incubation steps and advanced training in microscopy. Lastly, the small number of dogs included in this study represent a limitation. Further studies are needed to validate this technique in a larger population of clinical dogs and to determine its diagnostic and prognostic value in dogs with sepsis.

## Conclusion

NETs can be identified in septic fluids collected from clinical patients. The technique presented here can be a useful tool for the research of NETs and canine sepsis.

## Abbreviations

BAL: Bronchoalveolar lavage
cfDNA: Cell-free DNA
citH3: Citrullinated Histone H3
DPBS: Dulbecco’s phosphate buffered saline
ETW: Endotracheal wash
MPO: Myeloperoxidase
NETs: Neutrophil extracellular traps

## References

[1] Brinkmann V, Reichard U, Goosmann C, Fauler B, Uhlemann Y, Weiss DS, Weinrauch Y, Zychlinsky A (2004). Neutrophil extracellular traps kill bacteria. Science.

[2] Brinkmann V, Zychlinsky A (2007). Beneficial suicide: why neutrophils die to make NETs. Nat Rev Microbiol.

[3] McDonald B, Urrutia R, Yipp BG, Jenne CN, Kubes P (2012). Intravascular neutrophil extracellular traps capture bacteria from the bloodstream during sepsis. Cell Host Microbe.

[4] Czaikoski PG, Mota JM, Nascimento DC, Sonego F, Castanheira FV, Melo PH, Scortegagna GT, Silva RL, Barroso-Sousa R, Souto FO (2016). Neutrophil extracellular traps induce organ damage during experimental and clinical Sepsis. PLoS One.

[5] Chen R, Kang R, Fan XG, Tang D (2014). Release and activity of histone in diseases. Cell Death Dis.

[6] Dwivedi DJ, Toltl LJ, Swystun LL, Pogue J, Liaw KL, Weitz JI, Cook DJ, Fox-Robichaud AE, Liaw PC, Canadian Critical Care Translational Biology G (2012). Prognostic utility and characterization of cell-free DNA in patients with severe sepsis. Crit Care.

[7] Kawai C, Kotani H, Miyao M, Ishida T, Jemail L, Abiru H, Tamaki K (2016). Circulating extracellular histones are clinically relevant mediators of multiple organ injury. Am J Pathol.

[8] Jeffery U, Kimura K, Gray R, Lueth P, Bellaire B, LeVine D (2015). Dogs cast NETs too: canine neutrophil extracellular traps in health and immune-mediated hemolytic anemia. Vet Immunol Immunopathol.

[9] Li RHL, Ng G, Tablin F (2017). Lipopolysaccharide-induced neutrophil extracellular trap formation in canine neutrophils is dependent on histone H3 citrullination by peptidylarginine deiminase. Vet Immunol Immunopathol.

[10] Letendre JA, Goggs R (2017). Measurement of plasma cell-free DNA concentrations in dogs with sepsis, trauma, and neoplasia. J Vet Emerg Crit Care (San Antonio).

[11] Hamaguchi S, Akeda Y, Yamamoto N, Seki M, Yamamoto K, Oishi K, Tomono K (2015). Origin of circulating free DNA in Sepsis: analysis of the CLP mouse model. Mediat Inflamm.

[12] Hauptman JG, Walshaw R, Olivier NB (1997). Evaluation of the sensitivity and specificity of diagnostic criteria for sepsis in dogs. Vet Surg.

[13] Negoescu A, Labat-Moleur F, Lorimier P, Lamarcq L, Guillermet C, Chambaz E, Brambilla E (1994). F(ab) secondary antibodies: a general method for double immunolabeling with primary antisera from the same species. Efficiency control by chemiluminescence. J Histochem Cytochem.

[14] Narasaraju T, Yang E, Samy RP, Ng HH, Poh WP, Liew AA, Phoon MC, van Rooijen N, Chow VT (2011). Excessive neutrophils and neutrophil extracellular traps contribute to acute lung injury of influenza pneumonitis. Am J Pathol.

[15] Stelmaszynska T, Zgliczynski JM (1974). Myeloperoxidase of human neutrophilic granulocytes as chlorinating enzyme. Eur J Biochem.

[16] Papayannopoulos V, Metzler KD, Hakkim A, Zychlinsky A (2010). Neutrophil elastase and myeloperoxidase regulate the formation of neutrophil extracellular traps. J Cell Biol.

[17] Wang Y, Li M, Stadler S, Correll S, Li P, Wang D, Hayama R, Leonelli L, Han H, Grigoryev SA (2009). Histone hypercitrullination mediates chromatin decondensation and neutrophil extracellular trap formation. J Cell Biol.

[18] Leshner M, Wang S, Lewis C, Zheng H, Chen XA, Santy L, Wang Y (2012). PAD4 mediated histone hypercitrullination induces heterochromatin decondensation and chromatin unfolding to form neutrophil extracellular trap-like structures. Front Immunol.

[19] Thammavongsa V, Missiakas DM, Schneewind O (2013). Staphylococcus aureus degrades neutrophil extracellular traps to promote immune cell death. Science.

[20] Matthijsen RA, Huugen D, Hoebers NT, De Vries B, Peutz-Kootstra CJ, Aratani Y, Daha MR, Tervaert JWC, Buurman WA, Heeringa P (2007). Myeloperoxidase is critically involved in the induction of organ damage after renal ischemia reperfusion. Am J Pathol.

[21] Gould TJ, Vu TT, Swystun LL, Dwivedi DJ, Mai SH, Weitz JI, Liaw PC (2014). Neutrophil extracellular traps promote thrombin generation through platelet-dependent and platelet-independent mechanisms. Arterioscler Thromb Vasc Biol.

[22] Wright TK, Gibson PG, Simpson JL, McDonald VM, Wood LG, Baines KJ (2016). Neutrophil extracellular traps are associated with inflammation in chronic airway disease. Respirology.

